# Inherited Cardiomyopathies and the Role of Mutations in Non-coding Regions of the Genome

**DOI:** 10.3389/fcvm.2018.00077

**Published:** 2018-06-26

**Authors:** Oday F. Salman, Hebah M. El-Rayess, Charbel Abi Khalil, Georges Nemer, Marwan M. Refaat

**Affiliations:** ^1^Division of Cardiology, Department of Internal Medicine, Faculty of Medicine, American University of Beirut, Beirut, Lebanon; ^2^Department of Genetic Medicine, Weill Cornell Medical College, Doha, Qatar; ^3^Department of Biochemistry and Molecular Genetics, Faculty of Medicine, American University of Beirut, Beirut, Lebanon

**Keywords:** cardiomyopathy, hypertrophic cardiomyopathy, dilated cardiomyopathy, arrythmogenic cardiomyopathy, restrictive cardiomyopathy, spongiform cardiomyopathy, non-coding genome, mutations

## Abstract

Cardiomyopathies (CMs) are a group of cardiac pathologies caused by an intrinsic defect within the myocardium. The relative contribution of genetic mutations in the pathogenesis of certain CMs, such as hypertrophic cardiomyopathy (HCM), arrythmogenic right/left ventricular cardiomyopathy (ARVC) and left ventricular non-compacted cardiomyopathy (LVNC) has been established in comparison to dilated cardiomyopathy (DCM) and restrictive cardiomyopathy (RCM). The aim of this article is to review mutations in the non-coding parts of the genome, namely, microRNA, promoter elements, enhancer/silencer elements, 3′/5′UTRs and introns, that are involved in the pathogenesis CMs. Additionally, we will explore the role of some long non-coding RNAs in the pathogenesis of CMs.

## Introduction

Cardiomyopathies (CMs) are a group of cardiac pathologies caused by an intrinsic defect within the myocardium. By definition, CMs are not caused by hypertension, coronary artery disease, valvular heart disease, congenital heart disease, or active inflammation of the myocardium. There are 5 types of CMs: dilated cardiomyopathy (DCM), hypertrophic cardiomyopathy (HCM), restrictive cardiomyopathy (RCM), arrythmogenic right/left ventricular cardiomyopathy (ARVC/ALVC), and left ventricular non-compacted cardiomyopathy (LVNC).

DCM is characterized by global hypokinesia of the myocardium. Etiologies of DCM include excessive alcohol intake, Beri Beri, cocksackie virus, cocaine, Chagas disease, drugs (such as doxorubicin or daunorubicin), hemochromatosis, and pregnancy. However, the majority of cases are idiopathic. Several genes have been implicated in the pathogenesis of DCM. Most of the identified mutations affect cytoskeletal proteins that stabilize the contractile units of the myocyte. Other mutations involving functional defects in force-generating mechanisms (e.g., impairment of energy production in the mitochondria) have also been implicated. Such mutations in force-generation or force-transmission mechanisms causing sarcolemma/sarcomere disruption can lead to an impaired contraction. Genetic factors are thought to play a role in up to 30% of the cases of DCM (1–4). Some long non-coding RNA (lncRNAs) have been also found to play a role in the DCM phenotype pathogenesis (5).

HCM is the most common cause of sudden death in young adults. HCM is caused by a genetic defect, which is sporadic in 50% of cases. Familial cases are most commonly inherited in an autosomal dominant pattern. The genetic defects leading to HCM usually involve sarcomere disruption. Such defects trigger an alteration in gene expression that leads to compensatory hypertrophy of the heart. Abnormalities in sarcomere function result in upregulation of many growth factors and lnrnas and, eventually, to hypertrophy (6, 7).

RCM is caused by pathological infiltrations of the myocardium that lead to diastolic heart failure. Etiologies of RCM include radiation, amyloidosis, sarcoidosis, some glycogen storage diseases, and even metastasis to the heart. There is a familial form of RCM that mostly involves the RNNI3 gene coding for cardiac troponin I. Other familial forms involving different genes are less common.

ARVD is the second leading cause of sudden death in young adults after HCM. It is a genetically heterogeneous disorder most commonly inherited in an autosomal dominant manner. It is associated with mutations of cell-cell adhesion proteins; namely, desmosomal proteins like plakophilin-2, plakoglobin, desmoplakin (8–10) and is characterized by fibrofatty degeneration of the right ventricular myocardium (11, 12) Such a histologic change disrupts myocyte electrical conduction and increases the chance of re-entrant arrhythmias originating from the right ventricle. Manifestations can vary from isolated premature ventricular beat to fatal ventricular fibrillation (13).

LVNC (commonly called spongiform CM), like ARVD, it is a heterogeneous genetic disorder that results in the arrest of cardiac development during embryogenesis. Autosomal dominant, mitochondrial, and x-linked modes of inheritance have been reported in children; however the autosomal dominant pattern is the most predominant form. It is described grossly as having a 2-layer myocardium with many intraventricular trabeculations (14–16). Patients may present at any age with highly variable cardiovascular symptoms (17–21).

The role of genetics in CMs varies greatly in terms of penetrance, mode of inheritance and association. Below we will discuss the association of different non-coding mutations in CMs starting from intronic mutations followed by miRNA, 5′/3′ UTR, and promoter/enhancer element mutations (Table 1). Finally, we will explore the role of lncRNA in CM pathogenesis.

**Table 1:** Summary of the mutations in the non-coding genome involved in inherited cardiomyopathy.

	**HCM**	**DCM**	**ARVD**	**LVNC**	**RCM**
*MYBPC3*-c.506-2A>C	•				
*MYBPC3*-c.2308+3G>C	•				
*MYBPC3-* c.906–36G>A	•				
*MYBPC3*-c.906–1G>C	•				
*MYBPC3-* c.1224–2A>G	•				
*MYBPC3-* c.1224–19G>A	•				
*X25:* GAA Triplet repeat expansion (66–1,800 repeats)	•	•			
*DMD:*G>T at +1 position of 5′splice site at exon1-intron1 junction		•			
*DMD:* exon1 and muscle promoter deletion		•			
*PKP-2:* c.1378+1G>A			•		
^*^miRNA 1-2: rs9989532 A>G	•				
*DPMK:* CTG Triplet repeat expansion(≥50 CTG repeats)		•			
*RTN4:* 150bp TATC repeat polymorphism on 3′UTR		•			
*RTN4:* 127 bp CAA repeat polymorphism on 3′UTR		•			
*ISL1:* rs121913539 C>T		•			
^*^ISL1: rs71618117 G>C	•				
^*^ISL1: rs36216897 A>G	•				
^*^ISL1: rs116222082 G>A	•		•		
*TGF*β3: c.-36 G>A			•		
*TGF*β3: c. 1723 C>T			•		
^**^IKBL:−62AA		•			
^**^IKBL:−62AT		•			
^**^IKBL:−262AA		•			
^**^CCL2/MCP-1:−2518AA		•			
^**^CCL2/MCP-1:−2518AG		•			
^***^CCL2/MCP-1:−2518A		•			
^**^BAT1:−22CC		•			
^**^BAT1:−348CC		•			
*14-3-3*ε: c.-458 G>T				•	

* *Represents mutations with possible association to the corresponding cardiomyopathy, but further studies are required to determine a definite association*.

** *Represents a genotype associated with a corresponding cardiomyopathy instead of a variant (IKBL:−62AA for example resemble a homozygous genotype with an Adenine base at location−62 of the IKBL gene)*.

*** *Represents an allele associated with a cardiomyopathy instead of a mutational variant*.

## Intronic mutations

Approximately 10% of pathogenic mutations are intronic mutations that lead to defects in alternative splicing mechanisms (22) Myosin binding protein C3, dystrophin and plakophilin-2 genes exhibit many intronic point mutations that lead to disruption in alternative splicing mechanisms and thus insertions/deletions in the reading frame. Such insertions/deletions consequently lead to premature stop codons resulting in truncated proteins. Intronic triplet repeat expansion mutations are a less common type of intronic mutations associated with CMs. Such mutations, seen in the frataxin gene, cause a subsequent downregulation of frataxin level instead of a truncated protein.

### Myosin binding protein C3 (MYBPC3)

MYBPC3 is a sarcomeric thick filament associated protein with many isoforms. It is a physiological target of cAMP-dependent protein kinase. Its phosphorylation is involved in increasing actomyosin ATPase activity and thereby increasing crossbridge cycling and contractility (23). In a recent study that included 400 patients from southern Italy with inherited CMs, 20 genes known to be implicated in disease were analyzed (24). One-hundred thirty-six different mutations where found to be implicated, of which 11 were intronic. The study used *in silico* analysis (Alamut focus 0.9) with 5 different algorithms to analyze the consequences of different intronic mutations on messenger RNAs (mRNAs). They also used reverse transcription polymerase chain reaction (RT-PCR) to sequence the patient mRNA (obtained by tissue sampling, minigene analysis or ectopically expressed mRNA) to verify the *in silico* results. In the study, researchers focused on five intronic variants. The five variants that were analyzed were part of three genes: *MYBPC3, ACTC1* associated with HCM, and *SCN5A* associated with Brugada syndrome (24). *MYBPC3*-c.506-2A>C mutation resulted in the loss of the canonical site of splicing and the appearance of a new acceptor splice site 7 nucleotides downstream. In case of this mutation, the *in silico* analysis and the patients' mRNA analysis were in 100% agreement.

Concerning the other mutation involving the MYBPC3 gene *(MYBPC3*-c.2308+3G>C), according to the *in silico* approach, the score of the canonical donor splice site in all five algorithms was decreased (more than 50% in two of these algorithms). As for the minigene analysis of the mutated construct, there was a skipping of the *MYBPC3* 23rd exon. However, when the wild type (WT) minigene was transfected into the HEK293 cells, there was coexistence of the skipped transcript as well as the normal one. This indicated that the canonical donor splice site of intron 23 was innately weak in the HEK293 cells.

*In silico* analysis of the *SCN5A*-c.393-5C>A mutation showed the formation of a new splice site three nucleotides upstream of the canonical one. However, in the minigene method, there was skipping of exon 4. The discrepancy is likely because the WT consensus site is not clear yet, so the *in silico* approach becomes less reliable. Finally, the last two variants studied *MYBPC3*-c.906-7G>T and *ACTC1*-c.617-7T>C showed no changes in the alternative splicing of the RNA in neither 2 methods.

The *MYBPC3*-c.506-2A>C and *MYBPC3*-c.2308+3G>C mutations probably elicited their pathogenic potential in HCM through the frameshift they produced in the coding region of the gene as a result of defective splicing. This led to a C-terminal truncation of the protein.

*SCN5A*-c.393-5C>A mutation led to exon 4 skipping that resulted in an in-frame 30 amino acid (aa) deletion in the S1-DI transmembrane segment of the of the cardiac sodium channel alpha subunit. Since the voltage sensor domain needs the interaction of the S1-S3 segment with the S4 segment to function, the 30 aa in frame deletion in the S1-DI segment would lead to the loss of function of the sodium channel coherent with Brugada syndrome.

Another study also investigating mutations in introns flanking micro-exons in the *MYBPC3* gene included 250 unrelated HCM patients (25). Micro exons 10 (AGA), 14 (CAA) and 11 were screened for flanking intronic mutations. Seven mutations were identified in flanking intronic regions of micro-exons 10 and 14, while no intronic mutations were identified near micro-exon 11.

In order to identify which mutations resulted in aberrant alternative splicing of the *MYBPC3, in silico* analysis by three different software programs was performed. Reverse transcription PCR of peripheral blood leukocytes whole RNA was then performed, followed by automated sequencing, in order to verify the results of the *in silico* analysis. Only 4 out of the 7 mutations resulted in aberrant alternative splicing which ultimately led to a frameshift mutation in the coding region of the gene and a premature stop codon.

Two of the 4 mutations flanking exon 10 resulted in a premature stop codon: c.906–36G>A and c.906–1G>C. The G>A mutation led to the addition of 34 nucleotides (nts) from intron 9 to exon 10, which created a new acceptor splice site for exon 10. Eventually this resulted in a frameshift mutation and a premature stop codon in exon 12. The G>C mutation resulted in the disruption of the 3′ splice site whereby it was shifted 2 nts downstream of the original splice site. According to the RNA studies, this resulted in the exclusion of the first 2 nts from exon 10. This frameshift mutation also led to a premature stop codon in exon 12.

Coming to exon 14, 2 out of the 3 mutations identified in flanking introns were shown to affect alternative splicing: c.1224–2A>G and c.1224–19G>A. The A>G mutation led to a 4 nt deletion from the transcript, 3 of which from exon 14 and 1 of which at the most 5′ nt from exon 15. The ensuing frameshift resulted in a premature termination codon in exon 15. The G>A mutation caused the appearance of a denovo acceptor splice site that led to the extension of the transcript by 17 nts. Similarly, the resulting frameshift caused a premature termination of transcription in exon 15.

### Dystrophin protein

The dystrophin gene (DMD) traverses a 3 Megabase region on the X chromosome consisting of at least 79 exons (26–28). Dystrophin is a cytoskeletal protein that structurally links the myocytes cytoskeleton with the extra cellular matrix, providing muscular integrity and a mechanical signaling hub. It is expressed mainly in skeletal muscles, cardiac muscles, smooth muscles, and brain tissue, to a lesser degree (29). Many different isoforms of the dystrophin gene are present, for example muscle, brain, and purkinje isoforms. Some dystrophin defects may result in a familial form of dilated cardiomyopathy without skeletal muscle involvement like X-linked Dilated Cardiomyopathy (XLDC). A Substitution at the 5′ splice site of the dystrophin gene intron 1 had led to such a phenotype.

Two known syndromes caused by the mutation of the dystrophin gene are Duchenne muscular dystrophy (DMD) and Becker muscular dystrophy (BMD). Both syndromes are characterized by skeletal muscle involvement, with frequent myocardial involvement, as well. DMD is characterized mostly by deletions within the dystrophin gene that leads alterations in the dystrophin reading frame. BMD is a milder form of muscular dystrophy as compared to DMD. It is similarly often caused by deletions within the gene that result in a partially altered reading frame (30, 31).

The substitution of the G for a T at position +1 of the splice donor site of G100 T100 A/G95 A70 G80 T45 (where the numbers indicate the percentage of times with which the base is of consensus sequence) leads to a defect in mRNA maturation and eventually an abolition of the donor splice site between exon 1 and intron 1 (32). This results in the inclusion of the intron 1 sequence within the final transcript, thus disrupting the stability of the mRNA. As a result, there is an absence of the muscle isoform expression of the dystrophin gene (since the substitution was within the domain of the muscle isoform promotor). No arousal of a cryptic splice site was observed by such a substitution. Immunohistochemical staining of skeletal and cardiac muscles revealed that dystrophin expression in the heart was extremely diminished as compared to the slight decrease of dystrophin expression in skeletal muscles. Further immunohistochemical studies showed the expression of brain and purkinje dystrophin isoforms within skeletal muscle tissues but not in cardiac muscle tissue (33). This suggests the compensatory ability of the skeletal muscle to express different dystrophin isoforms when the muscle isoform is diminished. Such compensatory mechanisms were not observed in cardiac muscle tissue. The molecular mechanisms that explain the discrepancy in the compensatory ability of cardiac and skeletal muscle tissue are still unclear.

A similar phenotype was observed with the deletion of the first muscle exon, including the muscle promoter of the dystrophin gene (34, 35). Such a mutation also seemed to affect the cardiac muscle in a specific manner and led to a dilated cardiomyopathy phenotype. However, on skeletal muscle biopsy, there was some signs of mild myopathy. Immunohistochemical studies showed the presence of brain and purkinje dystrophin isoforms in skeletal muscles of affected patients whereas no such thing was observed in cardiac muscle. This also points toward the “compensatory ability” of the skeletal muscle compared to that of cardiac muscle.

### Plakophilin-2(PKP-2)

Plakophilin is a desmosomal protein involved in mechanical coupling of cardiac myocytes at intercalated disks. A heterozygous genotype with 1 plakophilin mutated allele is associated with most cases of ARVC (36). The mechanism by which such a mutation leads to ARVC (characterized by abnormal myocyte electrical conduction) is by the alteration of the distribution and the expression of connexin43. This is also seen in other common autosomal dominant desmosomal mutations. Connexin43 is part of the gap junctions present at the intercalated disks that insure proper electrical conduction between cardiac myocytes. Any disruption in their structures is believed to play a role in ARVC pathogenesis (37–39).

A novel intronic point mutation was identified in a patient with ARVC. The patient was found to be heterozygous for a c.1378+1G>A variant that is located at the upstream border of the splice site in intron 5 of the PKP-2 gene plakophilin (PKP-2: D460fsX464) (40). This point mutation led to the disruption of the splice site which eventually led to a truncated protein 464 aa residues long. Immunofluorescence and confocal microscopy techniques used on cardiac tissues obtained from patients with ARVC showed a disrupted distribution or expression of connexin43 in patients with PKP-2 mutations (37–39). In this particular patient, the PKP-2 head domain responsible for the formation of the desmosomal plaque is intact; however, the arm repeat domain is truncated. Consequently, more PKP-2 was found around the nucleus since this domain inhibits nuclear localization of PKP-2 (41). Moreover, since it's established that PKP-2 interacts with transcription factors (tfs) like TFIIIB and RNA polymerase III in the nucleus, we can say that such a localization is altering the gene expression of connexin43 (42).

### Frataxin protein

Although triplet repeat expansion mutations are uncommon among CMs, some diseases like Friedreich's ataxia and some muscular dystrophies are associated with DCMs.

Friedreich's ataxia is the most common inherited spinocerebellar degenerative disease (43). It is an autosomal recessive disorder resulting from the GAA triplet repeat expansion in intron 1 of the X25 gene in 96% of the cases and a point mutation or deletion with the triplet repeat expansion in 4% of cases (44, 45) The X25 diseased allele usually contains a 66–1,800 GAA triplet repeats in the first intron (45). This triplet repeat expansion will lead to the under-expression of the frataxin protein that functions in the formation of iron-sulfur clusters present in the mitochondria. Iron-sulfur clusters are involved in energy generation through the electron transport chain.

The frataxin protein also functions in quenching free iron in the mitochondria reducing the amount of reactive oxygen species (46–48). Intuitively, its downregulation would precipitate myocardial injury by many mechanisms. Myocardial involvement remains one of the most important prognostic factors in Friedreich's ataxia. A study on the longitudinal course of cardiomyopathy in Friedreich's ataxia patients recruited 28 affected patients and followed up with them for a median of 4.7 years (49, 50). The study relied on echocardiography, electrocardiography, doppler ultrasonography, MRI, and autopsy for patient assessment. Z-score statistical analysis was used to standardize differences in age and body size of the patients. They found that the predominant cardiomyopathy manifestation in these patients was HCM with a progressive decrease in ejection fraction (EF) with age (5 of the 13 HCM patients followed up showed a 55% decrease in EF with age). This study suggests a natural course from HCM to DCM in Friedreich's ataxia patients. There also appears to be a correlation between the length of the GAA triplet repeat on the shorter allele and the severity of the cardiomyopathy as well as the age of onset; the longer the GAA triplet repeat on the shorter allele, the more severe the cardiomyopathy, and the earlier the age of onset (51).

## miRNA mutations

MicroRNAs (miRNAs) are short (22 nucleotides) non-coding RNAs that bind to the mRNA transcript by partial complementarity and recruit a RNA-induced silencing complex (RISC) that leads to mRNA degradation and translational repression. miRNAs downregulate gene expression through binding to a short 6-nucleotide region in the 3′ UTR of the mRNA, leading to inhibition of translation or degradation of the target mRNA. In humans, it is estimated that miRNAs regulate approximately half of the transcriptome.

One of the important mediators of alteration of gene expression in different CMs are miRNAs. In fact, miRNA gene variants are thought to play a decent role in HCM pathogenesis. A study investigating this recruited 6 patients with HCM (compared with normal 6 donor hearts) caused by the *MYBPC3* mutation (52). In that study miRNA expression profiles were compared among *MYBPC3* mutated hearts and normal donor hearts. They used the whole genome approach and screened for expression of all miRNAs known (*n* = 664) of which 532 were expressed in at least 1 experimental heart. Expression of 13 different miRNAs revealed a unique signature in the HCM hearts (miR-181-a2, miR-184, miR-497, miR-204, miR-222, miR-96, miR34b, miR-383, miR-708, and miR-371-3p all were upregulated, and miR-10b, miR-10a, and miR-10b all were downregulated). Since these miRNA genes showed different expression profiles, it must mean that they regulate important mediators involved in the compensatory hypertrophic phenotype due to the mutation of MYBPC3. Since miRNA 204 was in the intronic region of the TRPM3 gene, the expression profiles of both the miRNA and the TRPM3 gene were analyzed and found to be co expressed. This suggests that miRNA 204 upregulates the expression of the TRPM3 gene. TRPM3 expresses the transient receptor potential cation channel correlated with increased calcium uptake of the myocardium and enhanced contractile function; it is a known mediator of HCM.

*In silico* analysis was performed to identify the target mRNAs of 9 out of the 13 different miRNAs identified (since the rest of the miRNA lacked targeting information in the database), based on sequence homology between 3′ UTRs and miRNA seed sequences. Four thousand forty-eight different mRNA targets were found for the 9-different miRNAs (including overlapping targets). A significant number of the target mRNAs were involved in the beta adrenergic signaling pathway that leads to dephosphorylation of downstream targets, like troponin I. This is a hallmark of HCM pathogenesis.

We can see here that the MYBPC3 mutations are probably causing a difference in expression of the specified miRNAs (and potentially much more) that in turn act on important mediators already known to be associated in the pathogenesis of HCM. This tells us that there's probably a panel of heritable miRNA gene variants (as discussed above) that could be potentiating or even causing HCM.

A later study investigated the presence of miRNA gene variants in HCM patients. In that study, 56 patients with HCM who were screened for the absence of mutations in 4 known genes associated with HCM (MYH7, *MYBPC3, TNNT2*, and *TNNI3*) and the absence of other comorbidities that usually result in diastolic dysfunction (like hypertension or valvular diseases) were tested for the presence of mutations in 11 different miRNA genes (53). Three known single nucleotide variants were identified on 3 different genes: rs45489294 (C>A with minor allele A) in the miRNA 208b gene, rs13136737 (C>A with minor allele A) in the miRNA 367 gene and rs9989532 (A>G with minor allele G) in the miRNA 1-2 gene. Two of these variants (miRNA 208b and miRNA 367) were found to have the same frequencies as in the control group. Pedigree analysis for the miRNA 1-2 in the family involved was performed. The variant was identified in three of the subject's offspring, however they did not have the HCM phenotype. This suggests that either the variant is associated with the HCM phenotype but with a certain penetrance or that this variant has the same frequency in the general population but it didn't appear in the control group due to the small size. The study was unable to proceed with the analysis of this variant due to the low sample size.

## 5′/3′ UTR mutations

Mutations in the 5′ and 3′ UTRs are also thought to contribute to the pathogenesis of some cardiomyopathies. While mutations in the 5′ UTR can lead to differential gene expression through a number of mechanisms, the mechanisms by which some 3′UTR mutations lead to differential gene expression are yet to be clearly illustrated. Dystrophia Myotonica 1 protein kinase and Reticulon-4 are genes found to be associated with DCM through mutations in the 3′UTR, whereas ISL-1 5′ UTR mutations are found to be associated with HCM and ARVC. Interestingly, defects in TGFβ3s 5′ and 3′ UTR regions are found to be implicated with ARVC.

### Dystrophia myotonica 1 protein kinase

Myotonic muscular dystrophy type 1 (DM1) is a disease caused by CTG triplet repeat expansion in the 3′ UTR region of the DPMK gene. DPMK is a serine-threonine kinase involved in intracellular signaling cascades mainly in rhabdomyocytes, cardiomyocytes and neurons. It is a regulator of many proteins that function in muscle contraction and relaxation, for example myosin phosphatase (54). An allele with a repeat number between 5 and 37 is considered stable. When the repeat number is between 38 and 49 the allele is unstable and is at risk of further repeat expansion during gametogenesis. A carrier of an allele with a repeat number of 50 or more invariably develops DM1 (55).

DM1 has a wide array of symptoms including muscle loss, weakness, rigidity, cataracts, and intellectual disability. Patients with DM1 usually develop dilated cardiomyopathy. The DMPK gene mutation can cause dilated cardiomyopathy secondary to DM1. A recent case study showed a probable association between de-novo CTG triplet repeat expansion in the DPMK gene and idiopathic dilated cardiomyopathy.

A 73 year-old woman with a permanent pacemaker for a complete AV block was diagnosed with idiopathic dilated cardiomyopathy (DCM) using echocardiography and endomyocardial biopsy of the right ventricular septum. The patient also had a history of cataracts and multiple miscarriages. PCR and southern blotting of peripheral blood mononuclear cells (PBMNC) for the DMPK gene were negative for the CTG triplet repeat expansion, so the patient did not have DM1. Small pool PCR techniques were used to reanalyze the PBMNCs of the patients and found a small percentage of them with the CTG expansion. The possibility of contamination of the patient's blood with a DM1 patient blood was eliminated; Moreover, the CTG triplet repeat expansion was confirmed by direct sequencing. Surprisingly, there was a significantly increased number of alleles carrying the CTG triplet repeat expansion in the cardiac muscle as opposed to PBMNCs. Additional screening of the SCA 1, 2, 3, 4, 6 and the DRPLA genes in the patient for CTG triplet repeat expansion was negative. This suggests that the CTG expansion occurring in the cardiac muscle at the DM1 locus is not due to a general defect in the system responsible for repeat stability all over the genome (56). This study suggests that a de-novo CTG triplet repeat expansion may be the cause of idiopathic DCM, despite a 10% prevalence of mutated cells in the heart (56).

### Neurite outgrowth inhibitor protein

Reticulon-4 (RTN4) gene is part of the reticulon family of genes. RTN4 gene encodes the nogo proteins present in 3 major isoforms: A, B, and C. Isoform A is highly expressed in the CNS, whereas isoform B is ubiquitously expressed in endothelial and vascular smooth muscle cell. It is associated with vascular remodeling. Isoform C is predominantly expressed in muscles. The bulk of studies involving the nogo proteins focus on their function in the central nervous system. Isoform A is an inhibitor of axonal regeneration and its loss of function is associated with the acceleration of CNS recovery after damage (57).

A pilot study in 2009 explored the association between 3′UTR TATC and CAA insertions/deletions in the RTN4 genes and DCM. In this study 159 unrelated patients with DCM where compared to 215 healthy subjects. A DNA extraction kit as well as PCR-polyacrylamide gel electrophoresis was used to identify the subjects' genotypes. (TATC_2_) and (TATC_1_) are different alleles resulting from the TATC insertion/deletion, manifested by different band sizes (150 and 147 bp respectively) on the polyacrylamide gel electrophoresis. The CAA alleles are manifested similarly (124 bp (CAA)_1_ and 127 bp (CAA)_2_). (CAA)_2_/(CAA)_2_, (CAA)_1_/(CAA)_2_, (CAA)_1_/(CAA)_1_, (TATC)_2_/(TATC)_2_, (TATC)_1_/(TATC)_2_, and (TATC)_1_/(TATC)_1_ are the genotypes that had their frequencies compared among the experimental and the control groups. (TATC_2_) was the only allele seen to have a significant (CI of 95%) difference in allele frequency among the 2 groups; moreover (TATC)_2_/(CAA)_2_ genotype was the only genotype similarly seen to have significant difference in genotype frequency among the 2 groups. In conclusion the (TATC_2_) allele and the (TATC)_2_/(CAA)_2_ genotype are associated with DCM. Thus, mutations in the 3′UTR of the RTN4 gene are associated with the DCM phenotype (58).

### Islet-1 transcription factor

Transcription factor islet-1 (ISL1) is one of the principal transcription factors involved in the development of the second myocardial lineage. It is expressed in the right ventricle, part of the atria, and a small portion of the left ventricle's inner wall. It also activates transcription of Mef2c tf by co-activating 2 enhancer regions with GATA, NKX2.5 and foxh1 tfs. It is a principal tf in heart development due to its spatial expression pattern and involvement in major cascades of heart development (59–61). Four hundred fifty-four unrelated patients where 296 had HCM, 78 DCM, 26 ARVC and 54 Emery–Dreifuss muscular dystrophy (EDMD) were recruited for a cohort study in order to identify new variants of the ISL1 locus by PCR screening. Eleven different variants were found on this locus. Of the 11 identified variants: c.-482G>C rs71618117, c.-302A>G rs36216897, c.-240G>A rs116222082, and c.-148C>T rs121913539 had an allele frequency of 0.5% in HCM, 0.2% in HCM, 0.7% in HCM/ARVC/EDMD and 0.1% in DCM respectively, but all had a 0% allele frequency in the control groups. This suggests a possible association of these variants with the corresponding cardiomyopathies. *In silico* analysis of variant c.-148C>T rs121913539 showed an 88% disease causing score and predicted the possible formation of a denovo donor splice site that leads to an abnormal ISL1 tf (62). Transforming Growth Factor Beta 3.

TGFβs are a superfamily of cytokines involved in cell growth, proliferation, differentiation and apoptosis. Some of them also stimulate of mesenchymal cells to produce ECM as well as inhibit metalloproteinase activity. Over expression of TGFβ3 *in vivo* has been shown to induce fibrotic change in a multitude of tissues (63, 64). A study shedding light on TGFβ3 as another gene which, upon mutation, is highly associated with the ARVC phenotype. The study was done on an ARVC1 family (38 members in 4 generations) and 30 unrelated individuals with clinical diagnosis of ARVC. Mutational screening by direct genomic DNA sequencing was done. Variant c.-36 G>A in the 5′UTR was found in all clinically affected individuals of the family as well as 3 asymptomatic relatives. Screening of the unrelated probands showed another variant in the 3′UTR (1723C
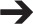
T) of the TGFβ3 gene. These variants were negative in all 300 controls. Luciferase reporter assays were used to test the effect of such 3′ and 5′ UTR mutations on TGFβ3 expression in C2C12 cells (since their TGFβ3 expression levels are similar to that of cardiac myocytes). The expression of TGFβ3 was twice that of wild type in the mutated gene constructs (*p* < 0.01) (65). The c.-36 G>A mutation in the 5′UTR causes an upregulation in TGFβ3 levels by substitution of an Arg to a His in a 88 aa peptide. This peptide is encoded by 1 of the 11 upstream open reading frames present in the 5′UTR of the TGFβ3 gene. This 88aa peptide that starts at the start codon (position−142) and ends in an overlap with exon1 of the TGFβ3 gene has an inhibitory role on TGFβ3 translation. This substitution renders this peptide less effective, and this upregulates TGFβ3 levels in cardiac myocytes (66, 67). The mechanism by which the 1723C
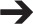
T variant in the 3′ UTR leads to upregulation of TGFβ wasn't clear. All patients in this study were screened for mutations in genes known to be associated with ARVC and turned out negative. It is still unclear whether the fatty deposition in the myocardium (part of the ARVC phenotype) is of primary or secondary consequence of TGFβ upregulation (68).

## Promotor/enhancer mutations

Promotor/enhancer mutations seem to be common among genes involved in the development of CCC in the chronic phase of Trypanosoma cruzi (T. cruzi) infection. Studies highlight the importance of different promotor/enhancer polymorphisms in NF-kappa-B inhibitor-like protein 1, C-C motif chemokine ligand 2/Monocyte chemoattractant Protein 1 and Spliceosome RNA Helicase BAT1. Chronic Chagas cardiomyopathy (CCC) is a condition caused 5–30 years after T. cruzi infection in a patient. It is characterized by myocarditis that eventually leads to fibrosis and DCM phenotype. T. cruzi has an acute phase and a chronic phase. Previous research has shown that only one-third of patients infected with T. cruzi develop CCC in the chronic phase of the infection (69). Promotor/enhancer variants in the 14-3-3 gene also seem to be important when it comes to LVNC.

### NF-kappa-B inhibitor-like protein 1

A study was conducted regarding susceptibility genes for CCC development in patients with the T. cruzi infection. The study compared polymorphisms in the IKBL gene promotor among 169 patients who developed CCC and 76 asymptomatic patients who did not. IKBL gene codes for an inhibitor of NFKB that is involved in the innate immune response. The study focused on 2 polymorphisms, the−62A/T and the−262A/G. It was shown that the−62AA and the−62AT genotypes were associated with a 3- and 2-fold increase in the chance of developing CCC respectively. The−262AA genotype also showed a 3-fold increase in the chance of developing CCC. When comparing severity of the cardiomyopathy among the patients with CCC (LVEF ≤ 40% and LVEF>40%) no association was found between the polymorphisms and the severity (70).

In a luciferase reporter assay for cells transfected with the different polymorphisms, it appears that the−62A is associated with a decreased expression of the IKBL gene, the inhibitor of NFKB. So such a polymorphism is associated with an increase in pro-inflammatory signals and disruption in regulation of inflammation. This leads to tissue damage consistent with CCC upon infection with T. cruzi (71) It appears that the−62T variant is part of an E-box sequence in the promotor region. This sequence is a binding site for the transcription factor USF1, so the−62A variant is apparently disrupting the USF1 binding and thus decreasing expression of IKBL. Similar studies for the−262A allele showed a consequential decrease in expression of IKBL as a result of the polymorphism (72).

### C-C motif chemokine ligand 2/monocyte chemoattractant protein 1

Another gene in which polymorphisms were associated with the development of CCC in the chronic phase of T. cruzi infected patients is the CCL2/MCP-1. This gene expresses a chemokine that is involved in the increased recruitment of macrophages upon a T. cruzi infection which leads to a better prognosis in the chronic course of infection; moreover it is thought to be also associated with the expression of the inducible nitric oxide synthase gene essential in T. cruzi clearance (73). A study looking into the presence of polymorphisms in the promotor region of CCL2 associated with CCC susceptibility included155 patients with CCC and 76 aymptomatic patients and analyzed the−2518 locus of the gene. It was found that genotypes AA and AG (at position−2518) are associated with a 4.1 and 2.7-fold increase in chance of CCC development upon T. cruzi infection as compared to the GG genotype. Comparison of allele frequency of−2518A among the two groups showed a 1.9 odds ratio for the A allele. Similarly to the previous study, LVEF of >40% or ≤ 40% was used to asses severity of the DCM in association with the−2518A/G polymorphisms. No statistically significant difference was noted for the association between the AA, AG genotype and the severity of the DCM as compared to the GG genotype (74).

### Spliceosome RNA helicase BAT1

BAT1 is a protein of the DEAD-box family of RNA dependent ATPases which mediates ATP hydrolysis during the splicing mechanism of the pre-mRNA. It's located in the MHC III region of chromosome 6. *In vitro* studies show that BAT1 acts by downregulating TNF-alpha and IL-6 cytokines (75). BAT1 promotor polymorphisms that seem to decrease promotor efficiency and thereby reduce BAT1 expression levels are associated with the CCC phenotype, since BAT1 modulates the levels of important proinflamatory molecules implicated in the development of CCC.

A study on 154 patients with CCC and 76 asymptomatic patients was done in order to analyze the association between the development of CCC and the gene promotor polymorphisms−22C/G and−348C/T. It was found that subjects homozygous for the−22C polymorphism showed a 5-fold increase in the risk of developing CCC compared with subjects having one or no copies of the−22C allele. Similarly for the−348C polymorphism, it was found that homozygotes were at two times the risk of developing CCC as compared to subjects with one or no copies of the−348C allele (76). The risk of development of CCC in T. cruzi infected patients appears to be multifactorial; identification of these factors may prove clinically significant as it will alter the care plan of patients predicted to acquire CCC in the future.

### 14-3-3 epsilon protein

14-3-3 is a family of phosphoserine and threonine binding scaffold adaptor proteins (77–80). There are 7 mammalian isoforms of the 14-3-3 family including the β, γ, ε, η, ζ, σ, and τ/θ where each isoform is encoded by a distinct gene (77, 78, 80–82). The 14-3-3ε isoform encoded by the YWHAE gene is involved in various processes and pathologies like Miller Dieke syndrome, periventricular heterotopias, corpus callosum hypoplasia, neuronal development and cardiac channel activity (77, 83–88) Recently it has been shown that mice lacking the YWHAE gene develop a left ventricular non-compaction cardiomyopathy (LVNC) phenotype with ventricular septal defects similar to the LVNC phenotype seen in humans (89).

A study was conducted to reveal the association between the YWHAE gene and the LVNC phenotype. Blood samples from 77 Japanese patients with LVNC were obtained and screened for variants. Seven variants were identified within the YWHAE gene, 4 within the promoter, 1 within an intron, 1 within the 3′UTR and 1 within exon 6. *In silico* analysis did not show any change in splicing patterns that resulted from any of these variants. Compared to the ethnicity matched normal controls, five of these variants showed no statistically significant difference in allele frequency. Of the two variants that showed statistical significance the intronic variant showed no alteration of splicing pattern *in silico*. The c.-458 G>T which is the remaining significant variant, resides on the C/EBP (CCAAT/enhancer binding protein) response element of the promoter. The patient in which the c.-458 G>T variant was identified suffered a familial LVNC and was shown to be negative for mutations involving either of the genes most commonly associated with LVNC (TAZ, DTNA, STN, SCN5A, CSX/Nkx2.5, FKBP12, and LDB3). *In vitro* studies using luciferase reporter essays revealed a 50% decrease in the promoter strength as a result of the mutation. *In silico* analysis revealed the enhanced affinity of the c.-458 G>T variant to the C/EBPβ isoforms. *In vitro* DNA binding assays verified the *in silico* results. As such, it appears that this mutation causes the enhanced affinity of the promoter to potent transcriptional suppressors which lead to a down regulation of the 14-3-3ε isoform (90).

## Long non-coding RNAs (lncRNAs) and cardiomyopathies' pathogenesis

According to the encyclopedia of DNA elements more than 90% of our genome is transcribed at some point in time where about 98% of it is in fact non-coding (91). lncRNAs are a type of non-protein coding RNA transcripts of variable sizes (extending from 200 nucleotides up to 100 kbp long) (88–90, 92). Studies implicate lncRNAs in gene imprinting, developmental and epigenetic regulation through various mechanisms like chromatin organization, transcriptional and post-transcriptional regulation. In fact, some lncRNAs have been shown to be exclusively localized in sub-nuclear compartments which highlights its activity in chromatin remodeling (91, 93–97) lncRNAs are involved in many aspects of gene regulation, so their alteration and contribution is expected in many pathologies.

A pilot study highlighted the role of lncRNAs in the pathogenesis of HCM. lncRNAs microarray analysis of myocardial tissue samples of 7 HCM patients were compared to controls for a minimum of 2-fold change (*P* < 0.05) in expression levels. Nine-hundred sixty-five upregulated and 461 downregulated lncRNAs were identified within the samples. This indeed suggested a role of lncRNAs in HCM pathogensis and possibly other cardiomyopathies of which mechanisms remain vague (7). Particularly, myocardial infarction–associated transcript is found to be differentially expressed in CCC patients as compared to other CM patients. Mice studies conveyed a possible role of metastasis-associated lung adenocarcinoma transcript 1 in diabetic cardiomyopathy pathogenesis. Uniquely “Myheart” a newly identified RNA cluster has been shown to have cardioprotective effects in stressed hearts.

### Myocardial infarction–associated transcript

Myocardial infarction–associated transcript (MIAT) is a lncRNA expressed mainly in the heart and developing brain tissue. Genome wide association studies correlated a MIAT haplotype to the risk of developing myocardial infarction (98). A study was done to characterize the correlation of MIAT expression with the inflammatory mediated dilated cardiomyopathy caused by chronic Chagas disease. Transcriptome analysis compared MIAT expression levels among 3 groups. Ten subjects with CCC, 14 subjects with idiopathic non-inflammatory mediated DCM and 7 controls had their heart tissue biopsy specimen analyzed. MIAT levels in patients with CCC were 3–49-folds greater than in the control group and 2–20-folds greater than in the DCM group. The increased expression of MIAT lncRNAs in CCC patients as compared to control and other non-inflammatory mediated DCM was verified by qRT-PCR in a larger sample of heart tissues. This indicates a role of MAIT in CCC pathogenesis though still through unclear mechanisms. Interestingly an extension of the study was done on mice where cardiac MIAT expression levels were measured at 15, 30, 45 days post T. cruzi infection. A correlation between MIAT expression levels and QTc interval prolongation was determined. This suggests an association between MIAT expression levels and CCC severity (5).

### Metastasis-associated lung adenocarcinoma transcript 1

Diabetic cardiomyopathy is known to be a ventricular dysfunction which is independent of coronary artery disease or hypertension. Pathological changes seen in a diabetic cardiomyopathy heart may include myocyte hypertrophy, myocardial fibrosis, and myofibril depletion (99).

A microarray study comparing the cardiac tissue transcriptome of DCM rats to healthy controls demonstrated the upregulation of the lncRNA metastasis-associated lung adenocarcinoma transcript 1 (MALAT1). This indicates the possible association of MALAT1 with diabetic cardiomyopathy pathogenesis. Another study compared the expression of inflammatory biomarkers like TNF alpha, IL-1beta, and IL-6 in diabetic cardiomyopathy mice in which MALAT1 was knockdown to a control group of diabetic cardiomyopathy mice where MALAT1 expression is intact. It appeared that MALAT1 knockdowns had a substantial decrease in expression of such inflammatory markers as compared to the controls (*P* < 0.05). Echocardiographic data was used to monitor cardiac structure and function including left ventricular end diastolic diameter (LVEDD), left ventricular end systolic diameter (LVESD), left ventricular fractional shortening (LVFS), and left ventricular ejection fraction (LVEF). LVFS and LVEF were found to be significantly increased in the MALAT1 knockdown group as compared with controls, whereas LVESD and LVEDD were found to be decreased in the MALAT1 knockdown group as compared to controls. This data suggests the possible role of the MALAT1 lncRNA in upregulating the expression of inflammatory markers that play a role in cardiac myocyte pathological changes seen in diabetic cardiomyopathy. Moreover, echocardiographic data suggest the contribution of MALAT1 in poor prognostic parameters (100).

### Mhrt

Recently, a new cluster of RNAs, Mhrt (Myheart), were discovered. Mhrt RNAs come from the alternative splicing of the myosin heavy chain 7 gene (Myh7) in cardiac myocytes. They range in size from 709 to 1147 nucleotides. Nuclear and cytoplasmic cellular extracts showed Mhrt to be exclusively localized in the nucleus. *In vitro* translation studies, coding substitution frequencies, and ribosomal profiling results showed no involvement of Mhrt in protein coding (101–103). So Mhrt RNAs are lncRNAs in cardiomyocyte nuclei. Pressure-overloaded hearts by transaortic constriction in mice showed a 46–68% downregulation in Mhrt expression. Moreover, a transgenic line of mice set to express Mhrt779 (the most abundant Mhrt with 779 nucleotides) in response to doxycycline were made (104). In comparison to the control group, the transaortic doxycycline treated transgenic mice revealed much less cardiac hypertrophy, fibrosis, ventricular dilatation and reduced fractional shortening. This indicates the protective role of Mhrt from pathological myocardial changes in response to stress. The experiment was repeated with treatment of doxycycline after 2 weeksof transaortic constriction (after the pathological changes had already started). Interestingly, similar cardioprotective role of Mhrt was shown (although to a lesser extent) in the experimental group as compared to the control group. This also shows the significance of sustained Mhrt repression in stressed hearts in order for pathological changes to take place.

A more recent study emphasizing Mhrt's cardioprotective role is investigating the use of obestatin with doxorubicin chemotherapy for less cardiotoxic outcomes (105). The hypothesis was based on a recent finding that obestatin can activate some anti-apoptotic signaling cascades and protect against ischemia induced myocardial injury and apoptosis.

Doxorubicin is an anthracycline cytotoxic drug used in the treatment of various neoplasms ranging from leukemias to metastatic carcinomas (106, 107) One of the most serious side effects of doxorubicin is cardiotoxicity that may lead to doxorubicin induced dilated cardiomyopathy (108). Obestatin is a ghrelin homolog with many anti-ghrelin effects (109). Doxorubicin and obestatin co-treated rats had a superior LV dp/dt_max_, LV peak systolic pressure and LV end diastolic pressure as compared to those treated with doxorubicin alone. This indicates the role of obestatin in decreasing the doxorubicin mediated cardiotoxic effects.

By comparing the Mhrt and Nrf2 cardiac myocyte expression levels in doxorubicin treated rats vs. that in doxorubicin-obestatin co-treated rats, it was found the Mhrt and Nrf2 expression levels were greatly decreased as a result of doxorubicin administration alone and were near normal levels as a result of co-treatment. Mhrt overexpressing transfected cardiomyocytes as compared to control cardiomyocytes upon doxorubicin administration showed less caspase 3 activity and significantly increased Nrf2 expression. Furthermore, the obestatin induced decrease in apoptotic activity as well as the increase in Nrf2 expression was abolished in Mhrt knockdown cardiomyocytes. This sequence of findings elucidates the mechanism behind which obestatin poses a cadioprotective function when co-administered with doxorubicin. Obestatin induces the expression of Mhrt (previously found to have cardioprotective roles) which in turn upregulates Nrf2. Nrf2 is a basic leucine zipper tf that modulates the expression of antioxidant proteins involved in many cardioprotective mechanisms (110). In conclusion Mhrt lncRNAs downregulation may be involved in the pathogenesis of DCM and HCM.

## Conclusion

The non-coding part of the genome is just recently being investigated for its involvement in various pathologies. Most of the high impact studies focused on heritable protein coding mutations. However, it is evident that mutations in parts of the genome like introns, 5′/3 UTR, miRNA, promotor/enhancer and possibly lncRNA affect overall protein structure, function, and expression to the same extent as coding mutations do, if not more drastically.

Intronic mutations of dystrophin, MYBPC3, plakophilin-2 and frataxin lead to drastic changes in the protein. Similarly, mutations in the promotor/enhancer regions of IKBL, CCL2/MCP1, BAT1, and 14-3-3 significantly change respective protein expression levels. Though the mechanisms are unclear, certain polymorphisms in the 5′/3′ UTRs of DM1, RTN4, ISL1, and TGFβ3 are associated with changes of protein function, structure, or expression.

In regards to miRNA, there is limited data regarding the association of certain variants with some CM phenotypes. However, microarray studies showed significant changes in miRNA expression profiles in the context of HCM. miRNA 1–2 variants implicated in HCM are plausible, but further studies are needed. The possible existence of lncRNA variants associated with CMs is well understated. MIAT overexpression was observed in CCC patients compared to patients with non-inflammatory mediated CMs. Mice studies further illustrated the roles of MALAT1 and myheart where MALAT1 was differentially expressed in diabetic cardiomyopathy mouse models and myheart showed to exhibit cardio protective functions in stressed hearts.

## Author contributions

All authors listed have made a substantial, direct and intellectual contribution to the work, and approved it for publication.

### Conflict of interest statement

The authors declare that the research was conducted in the absence of any commercial or financial relationships that could be construed as a potential conflict of interest. The reviewer AD and handling Editor declared their shared affiliation.
